# Drp1-mediated mitochondrial fission promotes cell proliferation through crosstalk of p53 and NF-κB pathways in hepatocellular carcinoma

**DOI:** 10.18632/oncotarget.11339

**Published:** 2016-08-17

**Authors:** Lei Zhan, Haiyan Cao, Gang Wang, Yinghua Lyu, Xiacheng Sun, Jiaze An, Zhenbiao Wu, Qichao Huang, Bingrong Liu, Jinliang Xing

**Affiliations:** ^1^ Department of Gastroenterology, Second Affiliated Hospital of Harbin Medical University, Harbin, 150086, China; ^2^ State Key Laboratory of Cancer Biology and Experimental Teaching Center of Basic Medicine, Fourth Military Medical University, Xi'an, 710032, China; ^3^ Department of Hepatobiliary Surgery, Xijing Hospital, Fourth Military Medical University, Xi'an, 710032, China; ^4^ Department of Clinical Immunology, Xijing Hospital, Fourth Military Medical University, Xi'an, 710032, China

**Keywords:** mitochondrial dynamics, cell proliferation, liver cancer, p53, NF-κB

## Abstract

Mitochondria are highly dynamic and undergo constant fusion and fission that are essential for maintaining physiological functions of cells. Recently, we have reported that increased mitochondrial fission promotes autophagy and apoptosis resistance in hepatocellular carcinoma (HCC) cell through ROS-mediated coordinated regulation of NF-κB and p53 pathways. However, little is known about the roles of mitochondrial dynamics in HCC cell proliferation, another key feature of cancer cells. In this study, we systematically investigated the functional role of mitochondrial fission in the regulation of HCC cell proliferation. Furthermore, the underlying molecular mechanisms were deeply explored. We found that, increased mitochondrial fission by forced expression of Drp1 promoted the proliferation of HCC cells both *in vitro* and *in vivo* mainly by facilitating G1/S phase transition of cell cycle. Whereas, Drp1 knockdown or treatment with mitochondrial division inhibitor-1 induced significant G1 phase arrest in HCC cells and reduced tumor growth in the xenotransplantation model. We further demonstrated that the proliferation-promoting role of Drp1-mediated mitochondrial fission was mediated via p53/p21 and NF-κB/cyclins pathways. Moreover, the crosstalk between p53 and NF-κB pathways was proved to be involved in the regulation of mitochondrial fission-mediated cell proliferation. In conclusion, our findings demonstrate that Drp1-mediated mitochondrial fission plays a critical role in the regulation of cell cycle progression and HCC cell proliferation. Thus, targeting Drp1-dependent mitochondrial fission may provide a novel strategy for suppressing tumor growth of HCC.

## INTRODUCTION

Hepatocellular carcinoma (HCC), the most common primary liver cancer, is the fifth most common malignant tumor worldwide and the third leading cause of cancer death [1]. Despite extensive application of targeted therapy, current treatment for advanced HCC is still not satisfactory [2]. Therefore, there have been continued interest and active research to find a novel strategy for suppressing tumor growth of HCC.

Cancer cells are characterized by excessive proliferation and thus tumor growth due to dysregulation of multiple cellular signaling pathway [3]. One such abnormality in cancer is p53 pathway, which is inactivated in the majority of human cancers due to downregulation or mutation. The key role of p53 as a tumor suppressor is to block cell cycle progression by activating the expression of downstream target genes, such as p21, which directly inhibits the activity of cyclin E/CDK2 complex and Rb and thus delay G1/S phase transition [4]. In addition, the NF-κB pathway, which transcriptionally controls a large set of cell cycle-related genes such as cyclin D and cyclin E and is frequently activated in many kinds of cancers, including HCC [5, 6]. Moreover, the crosstalk between these pathways becomes increasingly appreciated as an important mechanism operative during tumor progression [7, 8].

Mitochondria are highly dynamic and undergo constant fusion and fission that are essential for maintaining physiological functions of cells [9, 10]. The main mitochondrial fission and fusion proteins are members of the Dynamin family. Notably, dynamin related protein 1 (Drp1) and mitochondrial fission 1 (FIS1) are essential for mitochondrial fission. FIS1 protein is integrated into the outer mitochondrial membrane (OMM) and serves as a receptor for Drp1 which translocates from the cytosol to the OMM, where it forms a ring that drives fission of the organelle by a still unclear mechanism [11]. Deficiencies in the proteins regulating mitochondrial fission are associated with a number of human pathologies including neurodegenerative, neoplastic, endocrine, and cardiovascular diseases [12, 13]. Recently, cumulative evidence is beginning to reveal the close links between cancers and unbalanced mitochondrial dynamics. Several studies have reported that the expression of mitochondrial dynamic proteins such as Drp1 were dysregulated in human cancers of lung, brain and breast [14–17]. Very recently, we have reported that mitochondrial fission was frequently upregulated in HCC tissues, which significantly contributed to poor prognosis of HCC patients. Increased mitochondrial fission promoted the survival of HCC cells mainly by facilitating autophagy and inhibiting mitochondria-dependent apoptosis, which were mediated via the elevated ROS production and subsequent p53 degradation and NF-κB activation [18]. However, little is known about the roles of mitochondrial dynamics in HCC cell proliferation, another key feature of cancer cells. In this study, we systematically investigated the functional role of mitochondrial fission in the regulation of cell cycle progression and HCC cell proliferation. Furthermore, the underlying molecular mechanisms were deeply explored.

## RESULTS

### Upregualted Drp1 was significantly correlated with cell cycle-related genes at mRNA level in HCC tissues

**Figure 1:**
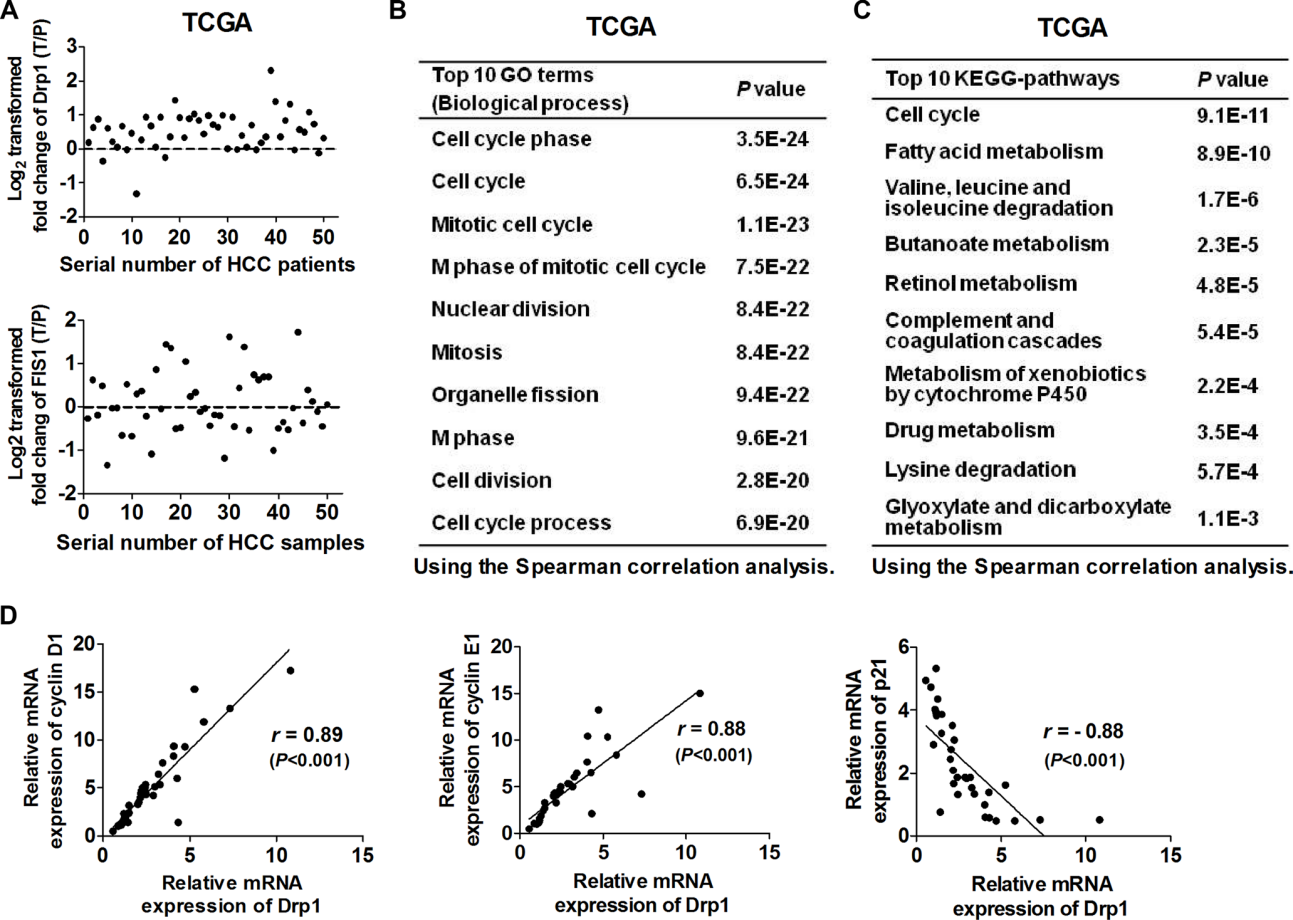
Expression of Drp1 was significantly correlated with the expression of cell cycle-related genes in HCC (**A**) Drp1 and FIS1 mRNA expression in paired HCC tissues were analyzed based on RNA-seq data from The Cancer Genome Atlas (TCGA). The relative expression ratio of tumor to peritumor was log_2_-transformed. The data was displayed according to serial patient ID number. T: tumor; P: peritumor. (**B** and **C**) Gene Ontology (GO) categories and Kyoto Encyclopedia of Genes and Genomes (KEGG) pathway enrichment analysis using the online DAVID tool for genes which were significantly correlated with Drp1. (**D**) Expression correlation between Drp1 and cell cycle related genes in HCC tissues were determined using Spearman correlation analysis. mRNA Expression levels of Drp1 and cell cycle related genes were detected by qRT–PCR in HCC tissues (*n* = 35).

In our previous study, the mRNA expression levels of Drp1 and FIS1 were analyzed by qPCR in 39 paired tissues from HCC patients [18]. To further investigate the Drp1 and FIS1 mRNA expression of HCC in a larger sample size, three public independent datasets from TCGA (50 paired HCC tissues) and GEO (GSE25097210, 211 paired HCC tissues; GSE36376, 182 paired HCC tissues) were analyzed using a data mining approach in the present study. Our results showed that Drp1 was remarkably upregulated at mRNA level in HCC tissues when compared with non-tumor tissues across three independent datasets. In contrast, FIS1 mRNA expression was not remarkably changed (Figure 1A and Figure S1A, S1B).These results confirmed our previous data that Drp1 was upregulated at the protein level in HCC tissues [18]. To further explore whether Drp1 is involved in the regulation of cell cycle in HCC cells, bioinformatic analysis was performed using DAVID online tool [19]. Gene Ontology (GO) categories and Kyoto Encyclopedia of Genes and Genomes (KEGG) pathway enrichment analyses showed that genes which were significantly correlated to Drp1 in mRNA expression were notably involved in cell cycle progression in all three datasets (Figure 1B, 1C, Figure S1C, S1D and Tables S4–S6). To further confirm these findings, we investigated the expression correlation of Drp1 and several key genes involved in cell cycle regulation in a panel of HCC tissues by qRT-PCR. Our data showed that the mRNA expression of Drp1 was positively correlated with the expression of cyclin D1 and cyclin E1 and negatively correlated with p21 (Figure 1D), suggesting that Drp1-mediated mitochondrial fission may play an important role in the regulation of cell cycle progression in HCC cells.

### Drp1-mediated mitochondrial fission promoted G1 to S cell cycle progression and proliferation of HCC cells

**Figure 2:**
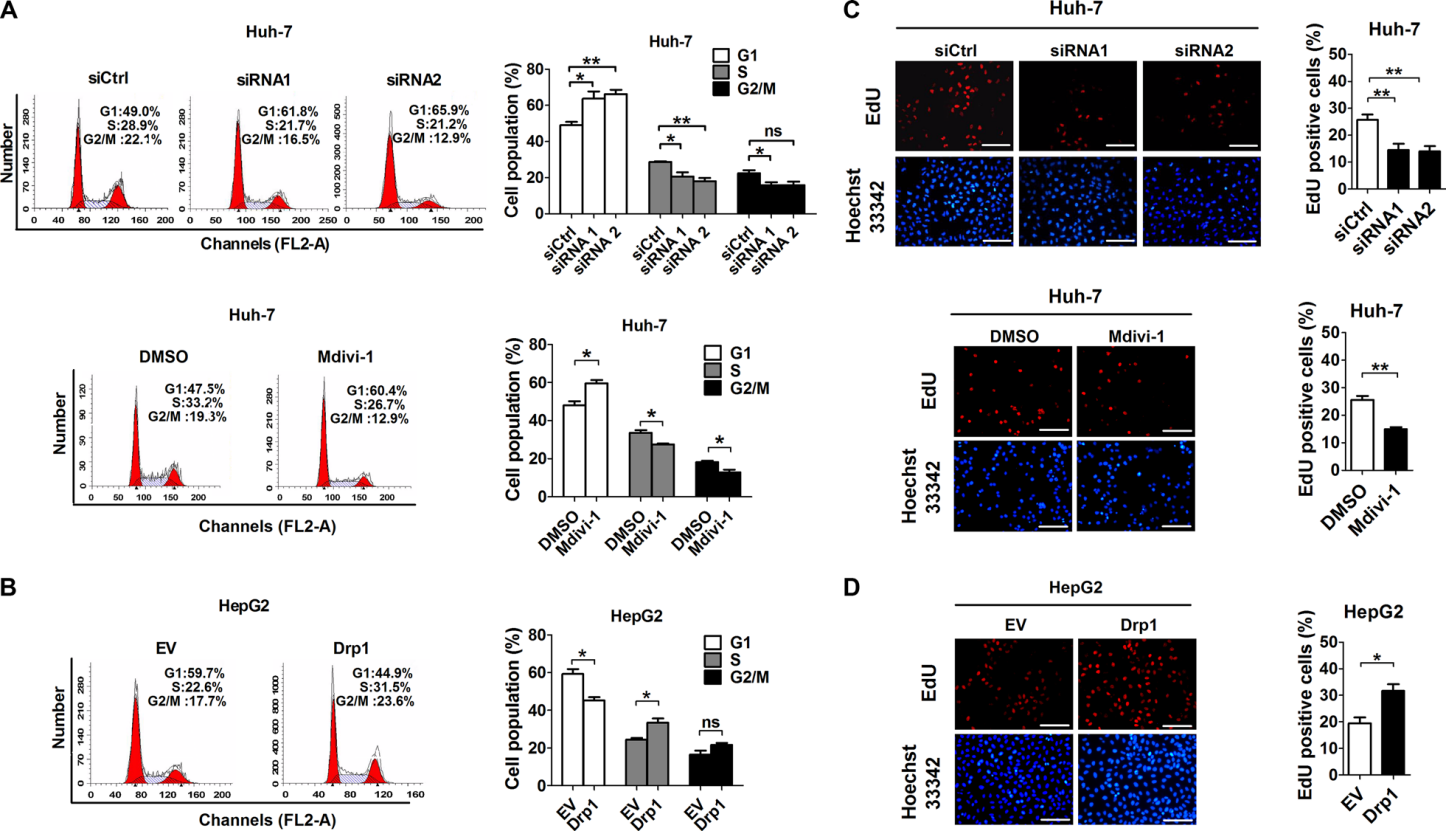
Drp1-mediated mitochondrial fission promoted proliferation of HCC cells *in vitro* (**A** and **B**) Cell cycle analysis by flow cytometry in Huh-7 and HepG2 cells 48 h after treatment with siRNA or expression vector. Huh-7 cells were also treated with Mdivi-1 (50 μM) or DMSO for 12 hours. (**C** and **D**) Cell proliferation was evaluated by EdU incorporation assay in Huh-7 and HepG2 cells with treatment as indicated. Scale bar, 50 μm. The data shown are the mean ± SEM from three separate experiments. *P < 0.05; **P < 0.01.

The endogenous expression level of Drp1 had been analyzed by qRT–PCR and Western blot in a panel of HCC cell lines in our previous study [18]. Additionally, the cell models with different Drp1 expression or activation (Figure S2A–S2C and [18]) were used to explore the effect of Drp1-mediated mitochondrial fission on cell cycle progression and cell proliferation in HCC. Quantitative analysis by flow cytometry indicated that Drp1 knockdown and Mdivi-1 treatment significantly increased the percentage of HCC cells in G1 phase of cell cycle. In contrast, Drp1 overexpression exhibited an opposite effect (Figure 2A, 2B and Figure S2D, S2E). Moreover, EdU incorporation assay revealed that HCC cells transfected with Drp1 siRNA or treated with Mdivi-1 had significantly less EdU incorporation than those in control cells. In contrast, HCC cells transfected with Drp1 expression vector had significantly more EdU incorporation than those transfected with empty vector (Figure 2C, 2D and Figure S2F, S2G). Taken together, all these results support the notion that Drp1-mediated mitochondrial fission promotes the proliferation of HCC cells by facilitating G1/S phase transition.

### Drp1-mediated mitochondrial fission promoted cell cycle progression through inhibiting p53 pathway

**Figure 3:**
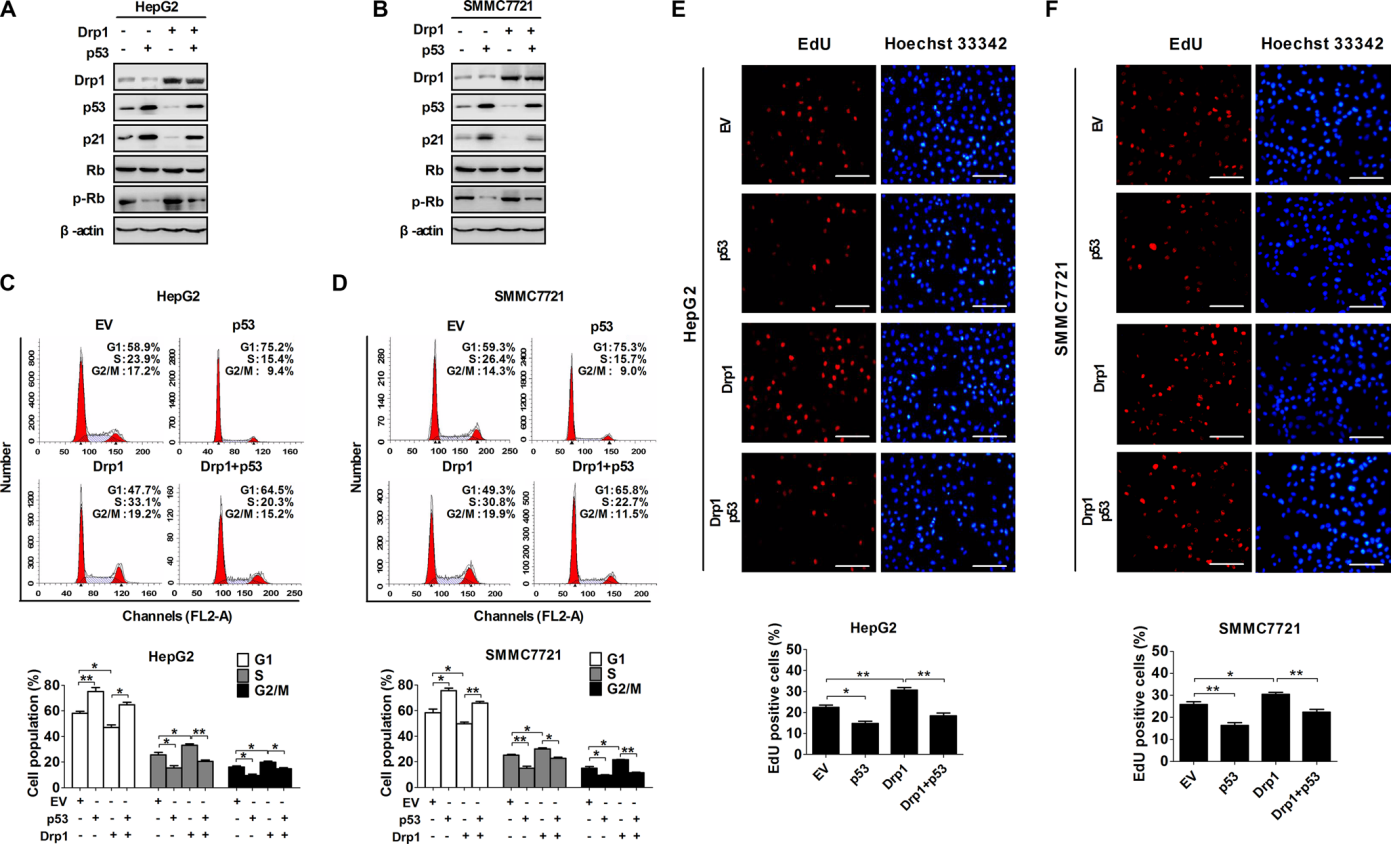
Drp1-mediated mitochondrial fission promoted cell cycle progression through p53 pathway (**A** and **B**) Western blot analyses for protein levels of Drp1, p53, p21, Rb, phosphorylated-Rb (p-Rb) in HepG2 and SMMC7721 cells with treatment as indicated. β-actin served as loading control. (**C** and **D**) Cell cycle analysis by flow cytometry in HepG2 and SMMC7721 cells 48 h after transfection with expression vector of Drp1 and/or p53. (**E** and **F**) Cell proliferation was evaluated by EdU incorporation assay in HepG2 and SMMC7721 cells as indicated in Panel (C and D). Scale bar, 50 μm. The results shown are the mean ± SEM from three separate experiments.

p53 is a crucial tumor suppressor that responds to diverse stress signals by orchestrating specific cellular responses, including transient cell cycle arrest, cellular senescence and apoptosis. Previously, we have demonstrated that increased mitochondrial fission inhibited apoptosis of HCC cells through p53 degradation mediated by ROS/Akt/MDM2 pathway. We thus further investigate whether cell cycle progression facilitated by mitochondrial fission is also in a p53-dependent way. Western blot analysis showed that both p53 and its target gene p21 (cyclin-dependent kinase inhibitor 1) were significantly decreased in both HepG2 and SMMC7721 cells with Drp1 overexpression, whereas phosphorylated-Rb was significantly increased when compared with those in control cells. Moreover, the effect of Drp1-mediated mitochondrial fission on the expression of cell cycle-related genes was reversed by exogenous p53 expression (Figure 3A, 3B and Figure S3A, S3B). Furthermore, inhibiting mitochondrial fission by Drp1 knockdown or Mdivi-1 treatment remarkably upregulated the expression of p53 and its target gene p21 in Bel7402 cells (Figure S4). We next investigated the functional role of p53 pathway in cell cycle progression regulated by Drp1-mediated mitochondrial fission. As expected, exogenous p53 expression considerably inhibited Drp1-mediated cell cycle progression and EdU incorporation (Figure 3C–3F). Thus, all these results indicate that Drp1-mediated mitochondrial fission regulates cell cycle progression by inhibiting p53 pathway in HCC cells.

### Drp1-mediated mitochondrial fission alternatively activated NF-κB/cyclins pathway to promote cell cycle progression

**Figure 4:**
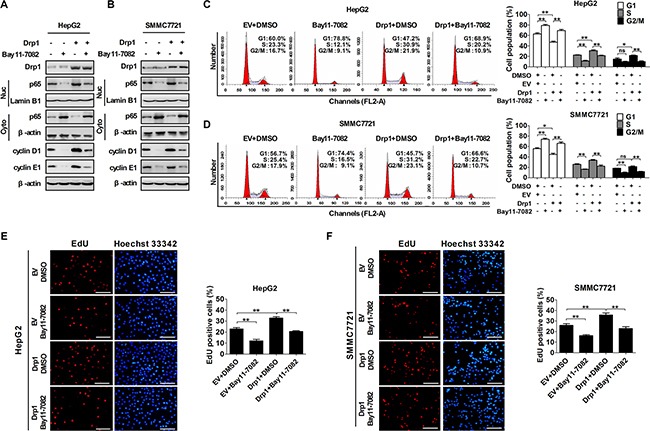
Drp1-mediated mitochondrial fission alternatively activated NF-κB/cyclin pathway to promote cell cycle progression (**A** and **B**) Western blot analyses for protein levels of Drp1, cyclin D1 and cyclin E1 in whole cells or p65 in cytoplasm and nucleus of HepG2 and SMMC7721 cells transiently transfected with Drp1 expression vector and then followed by treatment with NF-κB inhibitor Bay11-7082 (12.5 μM) for 12 hours. Empty vector or DMSO was used as negative control where appropriate. (**C** and **D**) Cell cycle analysis by flow cytometry in HepG2 and SMMC7721 cells 48 h after transfection with Drp1 expression vector. In the group with NF-κB inhibitor, cells were also treated with Bay11-7082 or DMSO (used as a control therapy) 12 h before analysis. (**E** and **F**) Cell proliferation was evaluated by EdU incorporation assay in HepG2 and SMMC7721 cells as indicated in Panel (C and D). Scale bar, 50 μm. The results shown are the mean ± SEM from three separate experiments.

Nuclear factor kappa B (NF-κB) has been implicated in the regulation of cell proliferation, transformation, and tumor development. Our previous study demonstrates that mitochondrial fission can promote transport of p65 (a key subunit of NF-κB) from cytoplasm to nucleus in HCC cells [18]. Therefore, we investigated the functional role of NF-κB pathways in cell cycle progression regulated by Drp1-mediated mitochondrial fission. As shown in Figure 4A, 4B and Figure S5A, S5B, nuclear transport of active p65 was inhibited by a specific NF-κB inhibitor Bay11-7082 in both HepG2 and SMMC7721 cells. Furthermore, inhibiting mitochondrial fission by Drp1 knockdown or Mdivi-1 treatment remarkably downregulated the nuclear expression of p65 and its target genes cyclin D1 and cyclin E1 in Bel7402 cells (Figure S4). Moreover, our data showed that Bay11-7082 treatment significantly decreased the expression of cell cycle-related key molecules cyclin D1 and cyclin E1 in HCC cells no matter whether Drp1 was force-expressed or not. Cell cycle analysis demonstrated that HepG2 and SMMC7721 cells treated with Bay11-7082 exhibited a significant accumulation of cells in the G1 phase (Figure 4C and 4D). As expected, Drp1-mediated EdU incorporation was also considerably inhibited by Bay11-7082 treatment (Figure 4E and 4F).

### Crosstalk of p53 and NF-κB pathways regulated by Drp1-mediated mitochondrial fission was essential for cell cycle progression

**Figure 5:**
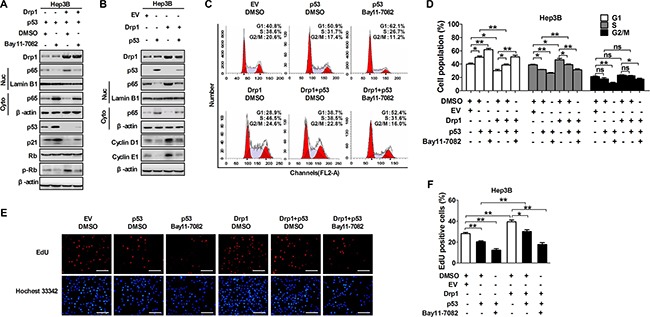
The crosstalk of p53 and NF-κB pathways regulated by Drp1-mediated mitochondrial fission was essential for cell cycle progression (**A**) Western blot analyses for protein levels of Drp1, p53, p21, Rb, phosphorylated-RB (p-Rb) in whole cells or p65 in cytoplasm and nucleus of Hep3B cells with treatment as indicated. (**B**) Western blot analyses for protein levels of Drp1, p53, cyclin D1 and cyclin E1 in whole cells or p65 in cytoplasm and nucleus of Hep3B cells transiently transfected with Drp1 and/or p53 expression vector as indicated. (**C** and **D**) Cell cycle analysis by flow cytometry in Hep3B cells 48 h after transfection with expression vector of Drp1 and/or p53. In the group with NF-κB inhibitor, cells were also treated with Bay11-7082 or DMSO (used as a control therapy) 12 h before analysis. (**E** and **F**) Cell proliferation was evaluated by EdU incorporation assay in Hep3B cells as indicated in Panel (C and D). Scale bar, 50 μm. The results shown are the mean ± SEM from three separate experiments.

Previously, we have demonstrated that crosstalk between p53 and NF-κB pathways induced by Drp1-mediated mitochondrial fission occurs in HCC cells and play an important role in apoptosis resistant and autophagy [18]. Therefore, in the present study, we explored whether Drp1-mediated cross-regulation of NF-κB and p53 pathways promoted HCC cell cycle progression and proliferation. The Hep3B HCC cell line (p53 gene deletion) was selected to be used in our study, which is a natural cell model to study the effect of p53 on multiple cell processes [20–22]. Our data showed that the inhibition of NF-κB pathway by Bay11-7082 treatment considerably upregulated the expression level of p53 and its target gene p21 and inhibited the level of p-RB in Hep3B cells with or without Drp1 overexpression when p53 expression vector was transfected (Figure 5A and Figure S6A). Similarly, the exogenous p53 expression exhibited a remarkable inhibitory effect on Drp1-mediated activation of NF-κB pathway and the expression of cyclin D1 and cyclin E1 in Hep3B cells (Figure 5B and Figure S6B). We further investigated the functional role of the interplay between p53 and NF-κB pathways in mitochondrial fission-mediated cell cycle progression. Our data showed that both the inhibition of NF-κB pathway by Bay11-7082 and exogenous p53 expression significantly inhibited G1/S transition and EdU incorporation (Figure 5C–5F). Importantly, the effect of Drp1-mediated mitochondrial fission on cell cycle progression and cell proliferation can be remarkably reversed by both treatments in Hep3B cells.

### Drp1-mediated mitochondrial fission promoted HCC cell proliferation *in vivo*

**Figure 6:**
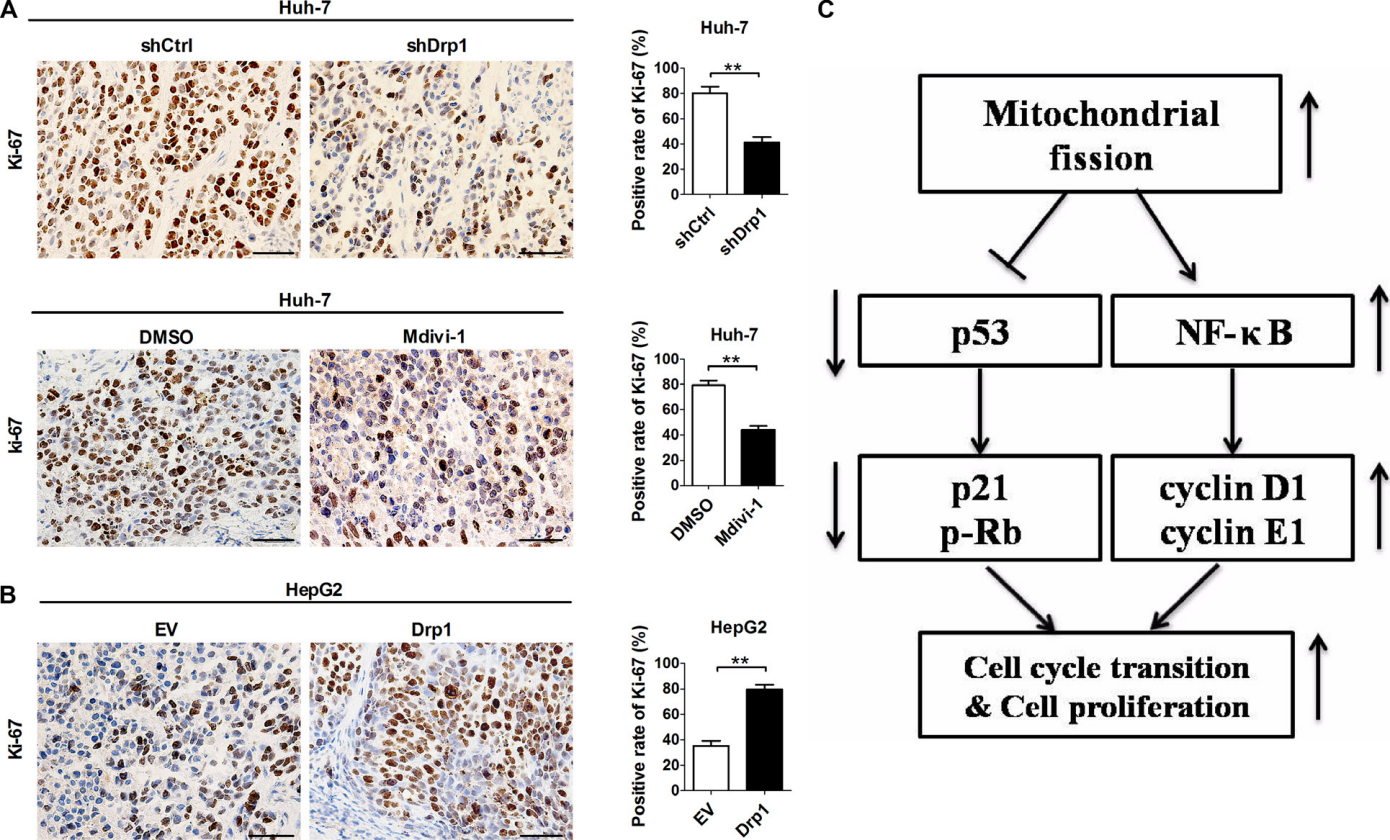
Drp1-mediated mitochondrial fission promoted proliferation of HCC cells *in vivo* (**A**) Representative immunohistochemical (IHC) staining images of Ki-67 in xenograft tumors developed from Huh-7 cells which were stably transfected with shRNA expression vector of Drp1 or treated with Mdivi-1 or DMSO as indicated. (**B**) Representative immunohistochemical (IHC) staining images of Ki-67 in xenograft tumors developed from HepG2 cells stably transfected with force-expression vector of Drp1 as indicated. Scale bar, 50 μm. (**C**) Schematic depicting the underlying mechanism of increased mitochondrial fission promotes cell cycle progression and proliferation in HCC. shDrp1: shRNA expression vector against Drp1. shCtrl: control shRNA. Drp1: expression vector encoding Drp1. EV: empty vector.

We next investigated the effect of altered expression or activity of Drp1 on tumor cell proliferation *in vivo* by constructing xenograft nude mice model using HCC cell lines with stable Drp1 knockdown, overexpression or inhibition (Figure S7A). Our results confirmed the previous report that, xenograft tumors developed from Huh-7 cells with stable knockdown of Drp1 or Mdivi-1 treatment exhibited a significant decrease in growth capacity when compared with their control xenograft tumors (*P* = 0.003 and *P* = 0.006, Figure S7B), whereas the growth capacity of xenograft tumors developed from HepG2 cells with stable Drp1 overexpression (Figure S7C) were much higher than corresponding control xenograft tumors (*P* = 0.003) [18]. Moreover, we have demonstrated that the fraction of Ki-67 (a nuclear proliferation antigen)-positive cells was significantly decreased in xenograft tumors developed from Huh-7 cells with stable Drp1 knockdown or Mdivi-1 treatment when compared with those in control xenograft tumors (Figure 6A). In contrast, forced expression of Drp1 significantly increased the fraction of Ki-67-positive cells in xenograft tumors developed from HepG2 cells (Figure 6B). Taken together, our data suggest that Drp1-mediated mitochondrial fission affects HCC cell proliferation *in vivo* and Drp1 selective inhibitor Mdivi-1 may be used as a promising novel therapeutic strategy for HCC.

## DISCUSSION

Mitochondrial morphology is regulated by the balance between fusion and fission, which is mediated by an evolutionarily conserved family of dynamin-related GTPases [23, 24]. In the present study, significant upregulation of Drp1 was found in HCC cells, which is essential for mitochondrial fission [18]. Consistent with our results, the upregulation of Drp1 and excessive fragmented mitochondria has also been reported in several other cancer types, including lung cancer [14], metastatic breast cancer [16], glioblastoma [25], and colorectal cancer [26]. Moreover, more comprehensive expression profiling of Drp1 is warranted in future studies.

In the present study, our data for the first time demonstrate that Drp1-mediated mitochondrial fission promotes cell cycle progression at G1 to S phase and subsequent proliferation in HCC cells. These results further support our interpretation that the increased ROS and its downstream signaling pathways induced by mitochondrial fission contributes to cell survival of cancer cells. Our findings therefore suggest a novel paradigm for cancer therapy, namely targeting mitochondrial networking. Consistently, a very recent study has showed that inhibition of mitochondrial fission causes cell cycle arrest in lung cancer cells, although the underlying mechanism is still unclear [14]. Moreover, Kashatus et al. have reported that Drp1 knockdown significantly inhibits the growth of xenograft tumor developed from pancreas adenocarcinoma cell line BxPC3 [27]. However, a previous study by Zhao J, et al. has reported the inconsistent results in breast cancer cells, indicating that Drp1 expression level has no significant effect on breast cancer cell cycle or cell viability. One possible explanation, as the author mentioned, is that the residual Drp1 protein after Drp1 knockdown may be still enough to maintain the cell cycle progression. Thus, choosing the right cancer cell lines as experimental models is critical for defining the pathological importance of Drp1 in cancer.

The tumor-suppressor protein p53 is a transcription factor that can induce either cell cycle arrest or apoptosis in many types of tumors [28–31]. Our previous study has demonstrated that PI3K/Akt pathway could induce the degradation of p53 protein in a MDM2-dependent manner [32]. In addition, we have previously demonstrated that Akt/MDM2/p53 pathway could be activated by mitochondrial fission-mediated ROS production in HCC cells [18]. However, whether the p53 degradation induced by mitochondrial fission affect cell cycle progression is still unclear. In the present study, our data showed that Drp1-mediated mitochondrial fission suppressed p21 expression through p53 degradation and thus promoted cell cycle progression. These observations support previous study that positive p21 expression in HCC is a predictor of better survival of patients after tumor resection [33]. Recent studies have showed the activation of NF-κB is one of the early key events involved in neoplastic progression of the liver and the inhibition of the NF-κB pathway decreases the expression of cell cycle regulator cyclin D and E, thus leading to G1/S arrest of cancer cells [34–36]. Moreover, the expression of cyclin D and cyclin E is frequently upregulated and considered to be useful prognostic indicators in many kinds of cancers, including HCC [5, 6, 37, 38]. We have previously showed that Akt/IKK/NF-κB pathway could be induced by mitochondrial fission-mediated ROS production in HCC cells. Therefore, in the present study, we further demonstrate that the activation of NF-κB pathway induced by Drp1-mediated mitochondrial fission also promotes cell proliferation through upregualting cyclin D1 and cyclin E1. In addition, we have also demonstrated that the crosstalk between NF-κB and p53 induced by Drp1-mediated mitochondrial fission is critical for proliferation of HCC cells, indicating a cooperative mechanism. However, whether mitochondrial fission regulates other signaling pathways involved in HCC cell proliferation is unclear. Further studies are necessary to define these complex mechanisms.

Our study focused on the role of Drp1 in cancer cell proliferation; however, it is likely that additional mediators and regulators may also play a key role in mitochondrial fission. Recent studies have demonstrated that, in addition to FIS1, mitochondrial fission factor (Mff), mitochondrial dynamics proteins of 49 and 51 kDa (MiD49 and MiD51, respectively) have been proposed to promote mitochondrial fission by recruiting the Drp1 [39]. Moreover, Guido C et al. have demonstrated that Mff-mediated mitochondrial fission induced glycolytic reprogramming in cancer-associated myofibroblasts, driving stromal lactate production, and early tumor growth [40]. However, in cancer cells, the function of these Drp1 receptors on proliferation is still unclear. Further studies are still needed.

Mdivi-1 is a derivative of quinazolinone, which selectively inhibits mitochondrial fission. It specifically inhibits Drp1 GTPase activity, blocks the self-assembly of Drp1 and causes the rapid and reversible formation of netlike mitochondria in both yeast and human cells [41, 42]. In the present study, our data clearly showed that Mdivi-1 treatment inhibited cell proliferation of HCC cells both *in vitro* and *in vivo*, which was consist with the effect of Drp1 knockdown. Several pilot studies have also shown that Mdivi-1 treatment could inhibit the cancer progression. For example, it has been reported that inhibition of Drp1-dependent mitochondrial fission by Mdivi-1 attenuated hypoxia-induced mitochondrial fission and migration in breast cancer and glioblastoma cell lines [25, 43]. Furthermore, Rehman et al. have recently shown that treatment with Mdivi-1 significant reduced the tumor growth of lung cancer *in vivo* [14]. However, it has also been reported that Mdivi-1 directly causes replication stress, mitochondrial dysfunction and subsequent cell apoptosis in a Drp1 independent way in multidrug resistant breast cells [44]. Therefore, the pharmacologic effect of Mdivi-1 on cancer treatment still need to be further study, considering its safety, specificity and tumor heterogeneity.

As summarized in Figure 6C, we demonstrate that Drp1-mediated mitochondrial fission facilitates cell cycle progression and cell proliferation through the crosstalk of p53 and NF-κB pathways. Moreover, our results confirmed the previous report that treatment with Mdivi-1 significant promoted tumor growth of HCC cells *in vivo*. Importantly, we further indicated the critical role of inhibition of Drp1 by Mdivi-1 on cell proliferation *in vivo*.

## MATERIALS AND METHODS

### Antibodies and reagents

The primary antibodies used in this study and their working concentration were listed in Table S1. The Drp1 inhibitor Mdivi-1 and NF-κB inhibitor Bay11-7082 were purchased from Sigma-Aldrich (St Louis, MO).

### Collection of public datasets and bioinformatic analysis

Three public datasets of mRNA expression data using paired HCC tissues were analyzed, including RNA-seq data from The Cancer Genome Atlas (TCGA) and microarray data of GSE36376 and GSE25097 from Gene Expression Omnibus (GEO) database. The detailed information was listed in Table S2. Expression correlation between Drp1 and cell cycle-related genes in HCC were determined using Spearman correlation analysis. Gene Ontology (GO) categories and Kyoto Encyclopedia of Genes and Genomes (KEGG) pathway enrichment analysis using the online DAVID tool (http://niaid.abcc.ncifcrf.gov/) was performed as previously described [19]. For all DAVID analyses, corrected *p*-values of less than 0.05 and fold enrichment of at least 1.5 were used as an empirical cut off for retrieving altered pathways.

### Cell culture and tissue collection

Human HCC cell lines HepG2, Huh-7, Bel7402, SMMC7721 and Hep3B (with p53 deletion) were routinely cultured. Forced expression and knockdown of target genes in HCC cells were carried out as previously described [18]. In addition, 35 human paired HCC tissue samples were obtained at Xijing Hospital affiliated with Fourth Military Medical University in Xi'an, China. None of these patients had received radiotherapy, chemotherapy or treated with sorafenib prior to surgery. The study was approved by the Ethic Committee of the Fourth Military Medical University and written informed consent was obtained from all participants.

### Quantitative real-time reverse transcription PCR (qRT-PCR)

Total RNA was extracted from cultured HCC cells or human HCC tissue samples using the TRIzol Reagent (Invitrogen). Genomic DNA digestion and reverse transcription were performed using the PrimeScript RT Reagent kit with gDNA Eraser (Takara, Dalian, China) according to the manufacturer's instructions. For the qRT-PCR analysis, cDNA were amplified using a SYBR Green PCR Kit (Takara, Dalian, China). The cycling parameters were 95°C for 15 s, 55°C for 15 s and 72°C for 15 s for 40 cycles. A melting curve analysis was then performed to check the specificity of PCR. The Ct value was measured during the exponential amplification phase. The relative expression level (defined as fold change) of the target gene was determined using a 2^−ΔΔCT^ method. GAPDH was used as internal control. The primers used in this study were listed in Table S3.

### Mitochondrial network imaging by confocal microscopy

The fluorescent dye MitoTracker green FM (Invitrogen) was used to monitor mitochondrial morphology in living cells according to the manufacturer's instructions. Olympus FV 1000 laser-scanning confocal microscope was used for cell imaging. For morphometric analysis, the length of mitochondria was measured using the Image J software (NIH, Bethesda, MD).

### Western blot analysis and immunohistochemical staining

Cell samples were lysed with RIPA buffer (Beyotime Inc., Nantong, China) and protein concentrations were measured using the micro BCA Assay (Pierce, Rockford, IL). The equal amounts (40 μg) of total protein was separated on 12% SDS–PAGE, and then transferred to polyvinylidene difluoride (PVDF) membrane (Millipore, Bedford, MA). The membranes were subsequently immunoblotted with the appropriate primary antibody. After extensive washings, the membranes were incubated with a secondary horseradish peroxidase-conjugated goat anti-mouse antibody (Pierce, Rockford, IL). Signals were detected using an ECL kit (Pierce, Rockford, IL) according to the manufacturer's instructions. All tissues were assessed by H&E staining to select suitable regions for immunohistochemical examination. Immunohistochemical staining was performed as previously described [32].

### Ethynyl deoxyuridine (EdU) incorporation assay

The proliferation of cells was analyzed using EdU incorporation assay kit (Ribobio, Guangdong, China) according to the manufacturer's instructions. Briefly, cells were incubated with 5 μM EdU in DMEM/RPMI-1640 (Hyclone) media supplemented with 10% FBS (Hyclone) for 2 h at 37°C. Then cells were fixed with 4% formaldehyde for 30 min at room temperature and treated with 0.5% Triton X-100 for 20 min for permeabilization. Then, cells were reacted with 1 × Apollo^®^ reaction cocktail (Ribobio, Guangdong, China) for 30 min. Subsequently, the DNA contents were stained with Hoechst 33342 for 30 min and visualized under a fluorescent microscope.

### Cell cycle analysis

Cell Cycle was determined by PI staining (BestBio, shanghai, China) following the manufacturers' instructions. Briefly, HCC cells seeded in 6-well plates were harvested and fixed in 70% ethanol and stored at 4°C overnight. Cells then were incubated with RNase at 37°C for 30 min, and stained with PI (1 mg/mL) for 30 min. Cell cycle analysis was performed by using a flow cytometry (Beckman, Fullerton, CA). The percentage of cells in the G1, S, and G2/M phases of cell cycle was determined by their DNA content.

### *In vivo* assays for tumor growth

Five-week-old male nude mice (BALB/c) were randomly divided into groups (seven mice/group). For tumor growth assay, 5 × 10^6^ cells mixed with matrigel were injected subcutaneously in the right and left flank of the mice, respectively. Tumor volume was double-blinded assessed each week after inoculation. After 4 weeks of injection, mice were euthanized and then the dissected tumors were weighed and analyzed. For Mdivi-1 treatment, two weeks after transplantation of tumor cells, Mdivi-1 or DMSO (negative control) was injected into each tumor xenograft twice a week at the dose of 0.75 mg/tumor. One month later, the mice were euthanized and then the dissected tumors were weighed and analyzed.

### Statistical analysis

Experiments were repeated three times, where appropriate. Data represent mean ± SEM. SPSS 17.0 software (SPSS, Chicago, IL) was used for all statistical analyses and *P* < 0.05 was considered significant. Unpaired *t* tests were used for comparisons between two groups where appropriate. Correlations between measured variables were tested by Spearman correlation analysis.

## SUPPLEMENTARY MATERIALS FIGURES AND TABLES








