# Supplementary material for: Drp1-mediated mitochondrial fission promotes cell proliferation through crosstalk of p53 and NF-κB pathways in hepatocellular carcinoma
Source: Oncotarget. 2016 Aug 17;7(40):65001–11. doi: 10.18632/oncotarget.11339 (PMC5323133; doi:10.18632/oncotarget.11339)
Supplement: Supplementary file 1 [file oncotarget-07-65001-s001.pdf]

Drp1-mediated mitochondrial fission promotes cell proliferation through crosstalk of p53 and NF-κB pathways in hepatocellular carcinoma

Supplementary Materials

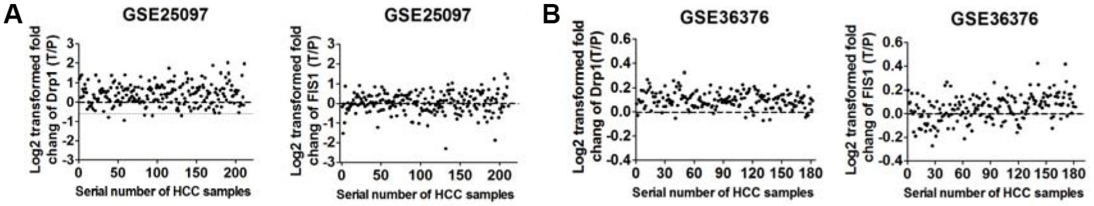

C

GSE25097

Top 10 GO terms (Biological process)	*P* value
Cell cycle	1.12E-21
Mitotic cell cycle	4.61E-21
Cell cycle phase	5.24E-21
M phase	2.20E-20
Organelle fission	2.73E-20
Cell cycle process	1.83E-19
M phase of mitotic cell cycle	4.50E-19
Nuclear division	2.36E-18
Mitosis	2.36E-18
Cell division	1.07E-14

Using the Spearman correlation analysis.

GSE36376

Top 10 GO terms (Biological process)	*P* value
Establishment of protein localization	6.20E-18
Mitotic cell cycle	1.10E-17
Protein transport	1.20E-17
Cell cycle	2.90E-16
Intracellular transport	8.10E-16
Protein localization	1.80E-15
RNA splicing	7.10E-15
mRNA processing	8.20E-15
RNA processing	1.40E-14
RNA splicing. via transesterification reactions with bulged adenosine as nucleophile	9.30E-14

Using the Spearman correlation analysis.

D

GSE25097

Top 10 KEGG-pathways	*P* value
Spliceosome	1.10E-14
ribosome	1.20E-08
Cell cycle	6.60E-06
DNA replication	6.10E-04
Mismatch repair	2.20E-03
proteasome	3.20E-03
Pathogenic Escherichia coli infection	3.30E-03
Huntington`s disease	3.80E-03
Pyrimidine metabolism	4.00E-03
Nucleotide excision repair	4.80E-03

Using the Spearman correlation analysis.

GSE36376

Top 10 KEGG-pathways	*P* value
Cell cycle	7.40E-07
Butanoate metabolism	2.60E-05
Fatty acid metabolism	5.00E-05
Valine, leucine and isoleucine degradation	1.80E-04
Complement and coagulation cascades	2.00E-04
Beta-Alanine metabolism	7.90E-04
Pyruvate metabolism	8.00E-04
Glycine, serine and threonine metabolism	8.80E-04
Drug metabolism	1.70E-03
PPAR signaling pathway	4.10E-03

Using the Spearman correlation analysis.

Supplementary Figure S1: Functional association and pathway analysis of Drp1 correlated genes in HCC. (**A** and **B**) Drp1 and FIS1 mRNA expression in paired HCC tissues were analyzed based on RNA-seq data of two largest public microarray mRNA expression datasets (GSE25097 and GSE36376) from GEO (Gene Expression Omnibus) database. The relative expression ratio of tumor to peritumor was log_2-transformed. The data was displayed according to serial patient ID number. T: tumor; P: peritumor. (**C** and **D**) Gene Ontology (GO) categories and Kyoto Encyclopedia of Genes and Genomes (KEGG) pathway enrichment analysis using the online DAVID tool for genes which were significantly correlated with Drp1.

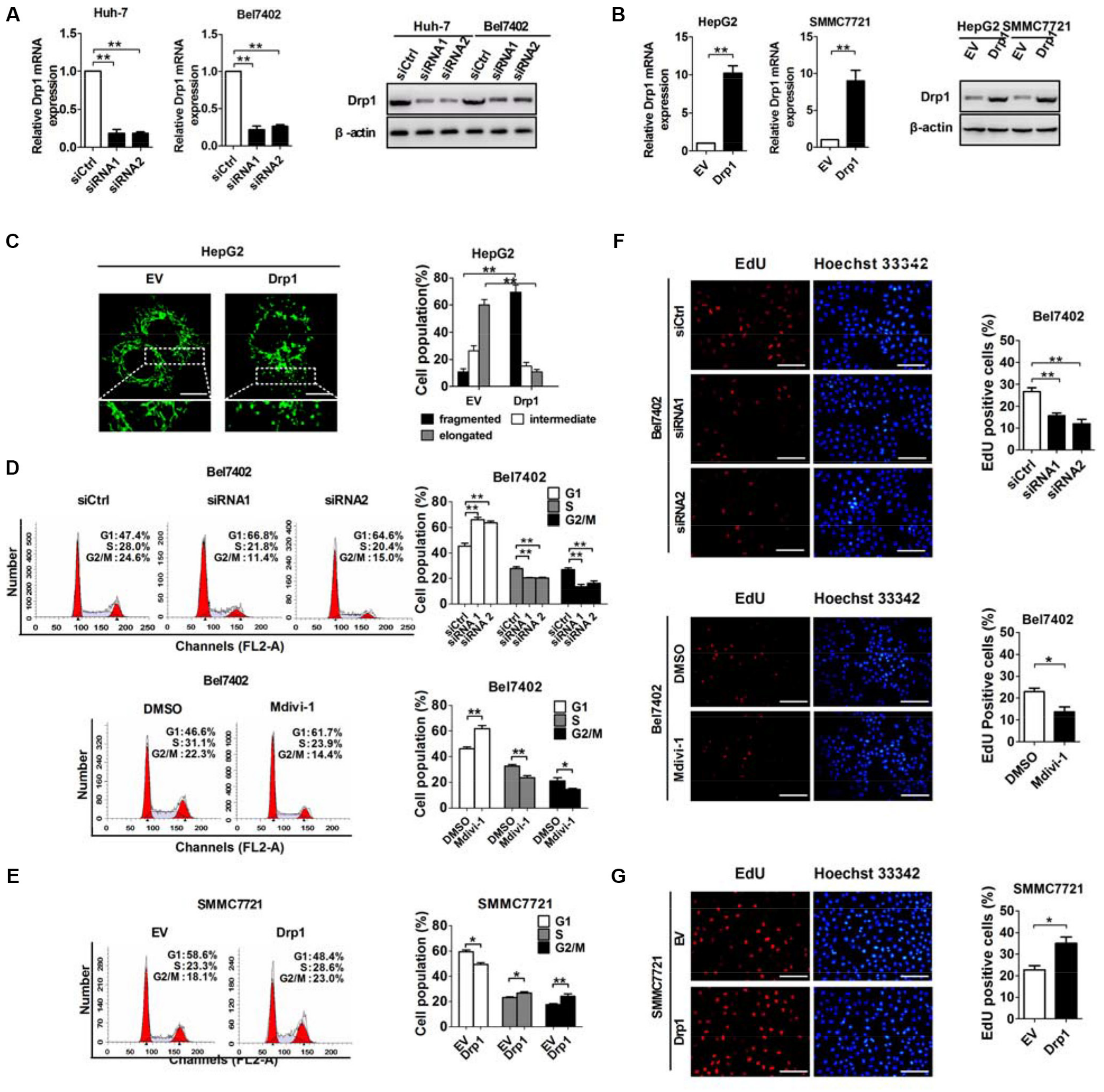

Supplementary Figure S2: Drp1-mediated mitochondrial fission promoted proliferation of HCC cells *in vitro*. (**A** and **B**) qRT–PCR and Western blot analyses for Drp1expression were performed in Huh-7, Bel7402, HepG2 and SMMC7721 cells 48 h after treatment with siRNA or expression vector as indicated. siRNA1 and siRNA2: siRNAs against Drp1. siCtrl: control siRNA. Drp1: expression vector encoding Drp1. EV: empty vector. (**C**) Confocal microscope analysis of mitochondrial network in HepG2 cells ($n = 50$ cells for each sample) 48 h after treatment with expression vector as indicated. Scale bars, 5 µm. The bottoms below are the enlarged views of the dotted rectangle areas in the upper images. (**D** and **E**) Cell cycle analysis by flow cytometry in Bel7402 and SMMC7721 cells with treatment as indicated. (**F** and **G**) Cell proliferation was evaluated by EdU incorporation assay in Bel7402 and SMMC7721 cells with treatment as indicated. Scale bar, 50 µm. The data shown are the mean ± SEM from three separate experiments. $*P < 0.05$; $**P < 0.01$.

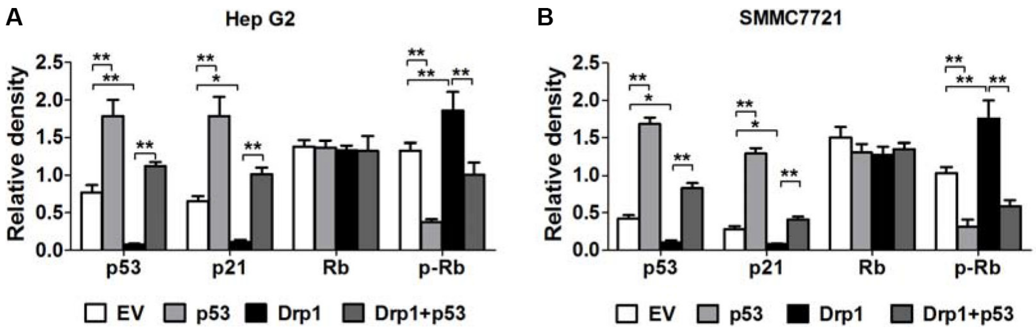

Supplementary Figure S3: Quantification of Western blot analysis for cell cycle-related genes in Figure 3A and 3B. The data shown are the mean ± SEM from three separate experiments. *$P < 0.05$; **$P < 0.01$.

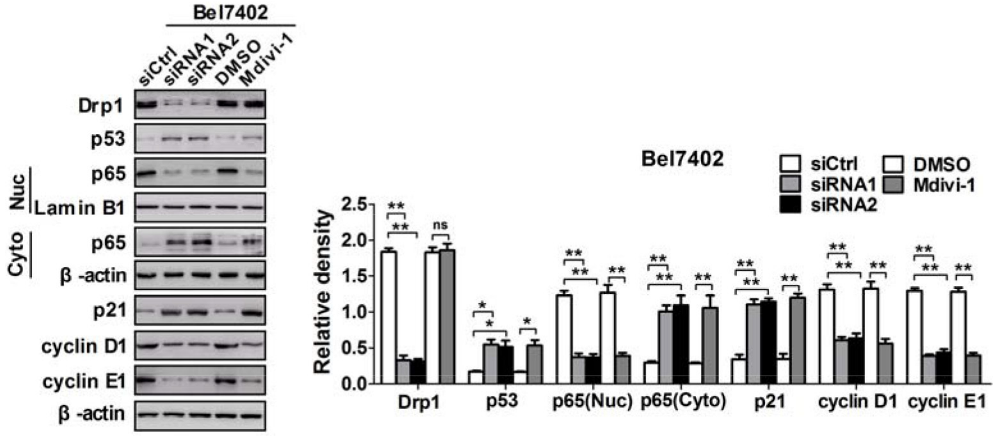

Supplementary Figure S4: Decreased mitochondrial fission inhibited cell cycle progression of HCC cells through regulating NF-κB and p53 pathways. Western blot analyses for protein levels of Drp1, cell cycle-related molecules p21, cyclin D1, cyclin E1 and p53 in whole-cells or p65 in cytoplasm and nucleus in Bel7402 cells with treatment as indicated. Quantification of Western blot analysis was shown in the right. The data shown are the mean ± SEM from three separate experiments. *$P < 0.05$; **$P < 0.01$.

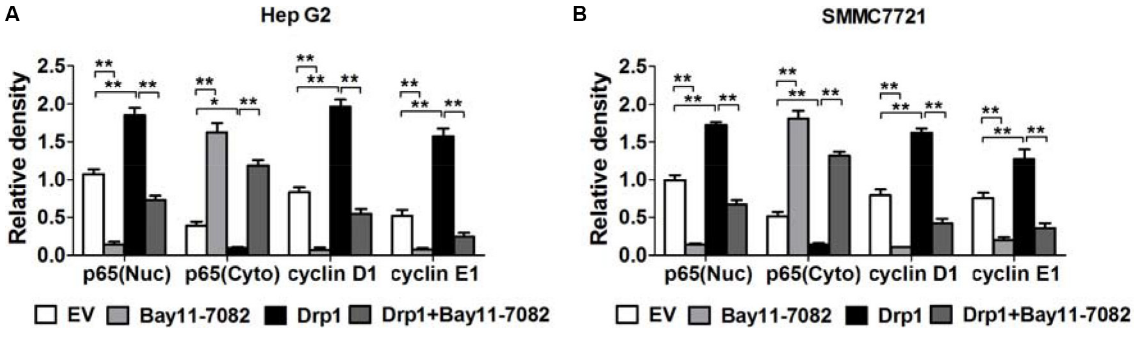

Supplementary Figure S5: Quantification of Western blot analysis for p65, cyclin D1 and cyclin E1 in Figure 4A and 4B. The data shown are the mean ± SEM from three separate experiments. *$P < 0.05$; **$P < 0.01$.

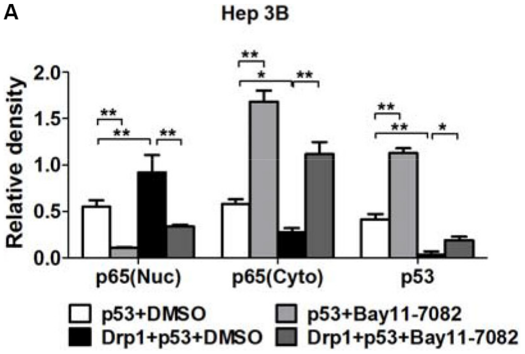

Supplementary Figure S6: Quantification of Western blot analysis for p53 and p65 in Figure 5A and 5B. The data shown are the mean ± SEM from three separate experiments. *$P < 0.05$; **$P < 0.01$.

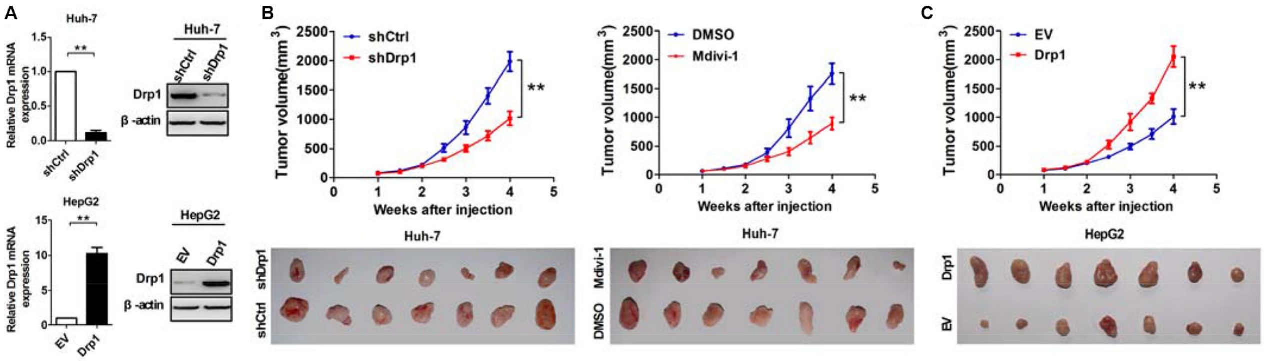

Supplementary Figure S7: Drp1-mediated mitochondrial fission promoted proliferation of HCC cells *in vivo*. (**A**) qRT–PCR and Western blot analyses for Drp1 expression were performed in Huh-7 and HepG2 cells stablely transfected with shRNA or force-expression vector of Drp1 as indicated. (**B** and **C**) Tumor growth curves of subcutaneous xenograft tumor model developed from HCC cells which were stably transfected with shRNA or force-expression vector of Drp1 as indicated (both $n = 7$). Huh-7 cells tumor-bearing mice were also treated with Mdivi-1 (0.75 mg/tumor) or DMSO by intratumor injection. Tumor size, including tumor length (L) and width (W), was measured using vernier calipers twice a week from day 7 after transplantation. The tumor volumes were calculated according to the formula $(L \times W^2)/2$ and presented as mean ± SEM. Tumors from sacrificed mice were dissected 4 weeks after transplantation and also shown in lower panel. shDrp1: shRNA expression vector against Drp1. shCtrl: control shRNA. Drp1: expression vector encoding Drp1. EV: empty vector.

Supplementary Table S1: Primary antibodies used for Western blot and immunohistochemistry

Antibody	Company (Cat. No.)	Working dilutions
Drp1	abcam (ab56788)	WB: 1/800
β-actin	Beijing TDY BIOTEC (TDY051C)	WB: 1/3000
p65	abcam(ab7970)	WB: 1/1000
Lamin B1	Beijing TDY BIOTEC (TDY049)	WB: 1/2000
p53	Cell Signaling (#9282)	WB: 1/500
p21	Proteintech (10355-1-AP)	WB: 1/1000
Rb	Proteintech (17218-1-AP)	WB: 1/1000
p-Rb	SAB (11132)	WB: 1/500
cyclin D1	Proteintech (60186-1-Ig)	WB: 1/2000
cyclin E1	Proteintech (11554-1-AP)	WB: 1/1000
Ki-67	ZSGB-BIO (ZA-0502)	IHC: 1/10000

Supplementary Table S2: Public datasets used for bioinformatic analysis in the study

Data source	Platform	Probes/Genes	Paired HCC Sample No.	Patient Ethnicity	Etiology	Source URL
TCGA	Illumina HiSeq_ RNASeqV2	- /20531	50	Asian, white, black or African american, American indian or alaska native	-	http://cancergenome.nih.gov
GEO (GSE36376)	Illumina HumanHT-12V4.0 Expression beadchip	47324 /18077	182	Korean	-	http://www.ncbi.nlm.nih.gov/geo/query/acc.cgi?cc=GSE36376
GEO (GSE25097)	Rosetta/ Merck Human RSTA Affymetrix1.0	37634 /18077	211	Chinese	HBV	http://www.ncbi.nlm.nih.gov/geo/query/acc.cgi?acc=GSE25097

Supplementary Table S3: Sequence of primers

Primers used in q-PCR analysis		
Drp1	forward primer	GGAGACTCATCTTTGGTGAAGAG
	reverse primer	AAGGAGCCAGTCAAATTATTGC
p21	forward primer	CGATGGAACTTCGACTTTGTCA
	reverse primer	GCACAAGGGTACAAGACAGTG
cyclin D1	forward primer	GCTGCGAAGTGGAAACCATC
	reverse primer	CCTCCTTCTGCACACATTTGAA
cyclin E1	forward primer	AAGGAGCGGGACACCATGA
	reverse primer	ACGGTCACGTTTGCCTTCC
GAPDH	forward primer	GGAGCGAGATCCCTCCAAAAT
	reverse primer	GGCTGTTGTCATACTTCTCATGG

Supplementary Table S4: Drp1 correlated genes in GSE25079. See Supplementary_Table_S4

Supplementary Table S5: Drp1 correlated genes in GSE36376. See Supplementary_Table_S5

Supplementary Table S6: Drp1 correlated genes in TCGA. See Supplementary_Table_S6
